# Flow-mediated-paradoxical vasoconstriction is independently associated with asymptomatic myocardial ischemia and coronary artery disease in type 2 diabetic patients

**DOI:** 10.1186/1475-2840-13-20

**Published:** 2014-01-15

**Authors:** Minh Tuan Nguyen, Isabelle Pham, Paul Valensi, Hélène Rousseau, Eric Vicaut, Christelle Laguillier-Morizot, Alain Nitenberg, Emmanuel Cosson

**Affiliations:** 1 AP-HP, Jean Verdier Hospital, Department of Endocrinology-Diabetology-Nutrition and Paris 13 University, CRNH-IdF, CINFO, Avenue du 14 juillet, Hôpital Jean Verdier, 93143, Bondy Cedex, France; 2 Sorbonne Paris Cité, UMR U557 INSERM/U11125 INRA/CNAM/Université Paris13, Unité de Recherche Epidémiologique Nutritionnelle, Bobigny, France; 3 Department of Clinical Physiology, AP-HP, Jean Verdier Hospital, Bondy, France; 4 Paris 13 University, Sorbonne Paris Cité, UFR SMBH, EA 2363 « Réponses cellulaires et fonctionnelles à l’hypoxie », Bobigny, France; 5 AP-HP, Unit of Clinical Research, Lariboisière Hospital, Paris 7 University, Paris, France; 6 Department of Biochemistry, AP-HP, Jean Verdier Hospital, Bondy, France

**Keywords:** Silent myocardial ischemia, Flow-mediated dilation, Asymptomatic coronary artery disease, Diabetes

## Abstract

**Background:**

To investigate whether flow-mediated dilation (FMD) impairment, which precedes overt atherosclerosis, is associated with silent myocardial ischemia (SMI) and asymptomatic coronary artery disease (CAD) in type 2 diabetes.

**Methods:**

Forearm FMD was measured by ultrasonography in 25 healthy control, 30 non-diabetic overweight or obese patients and 118 asymptomatic type 2 diabetic patients with a high cardiovascular risk profile. SMI (abnormal stress myocardial scintiscan and/or stress dobutamine echocardiogram) and CAD (coronary angiography in the patients with SMI) were assessed in the diabetic cohort.

**Results:**

FMD was lower in diabetic patients (median 0.61% (upper limits of first and third quartiles -1.22;3.2)) than in healthy controls (3.95% (1.43;5.25), p < 0.01) and overweight/obese patients (4.25% (1.74;5.56), p < 0.01). SMI was present in 60 diabetic patients, including 21 subjects with CAD. FMD was lower in patients with SMI than in those without (0.12% (-2.3;1.58) vs 1.64% (0;3.69), p < 0.01), with a higher prevalence of paradoxical vasoconstriction (50.0% vs 29.3%, p < 0.05). FMD was also lower in patients with than without CAD (-1.22% (-2.5;1) vs 1.13% (-0.4;3.28), p < 0.01; paradoxical vasoconstriction 61.9% vs 34.4%, p < 0.05). Logistic regression analyses considering the parameters predicting SMI or CAD in univariate analyses with a p value <0.10 showed that paradoxical vasoconstriction (odds ratio 2.7 [95% confidence interval 1.2-5.9], p < 0.05) and nephropathy (OR 2.6 [1.2-5.7], p < 0.05) were independently associated with SMI; and only paradoxical vasoconstriction (OR 3.1 [1.2-8.2], p < 0.05) with CAD. The negative predictive value of paradoxical vasoconstriction to detect CAD was 88.7%.

**Conclusions:**

In diabetic patients**,** FMD was independently associated with SMI and asymptomatic CAD.

**Trial registration:**

Trial registration number NCT00685984.

## Background

Type 2 diabetes is associated with a high prevalence of coronary artery disease (CAD) and with a 2- to 4-fold increase in silent myocardial ischemia (SMI) as compared with the non diabetic population [1,2]. SMI has been reported in 7 to 65% of the diabetic population [3], this prevalence increasing with male gender, ageing, longer duration of diabetes and the presence of additional cardiovascular risk factors, nephropathy, retinopathy and peripheral or carotid occlusive arterial disease [1-4]. SMI is a strong predictor of future coronary events and premature death [5,6], providing a significant additional value compared to routine cardiovascular risk assessment [7].

Endothelial dysfunction is an early phenomenon during diabetic atherogenesis [8,9] and has been associated with a poor cardiovascular prognosis in the diabetic population [10-12]. Therefore, peripheral endothelial dysfunction is considered as an integrator of cardiovascular risk. The association between endothelial and smooth muscle dysfunction evaluated by flow-mediated dilation (FMD) and SMI in patients with type 2 diabetes has been previously studied. Some authors did not find any association but reported a high negative predictive value for SMI when the endothelial function was normal [13], whereas others reported a higher prevalence of SMI in the patients with abnormal FMD [14]. However, these series included a limited number of subjects, whose *a priori* cardiovascular risk was lower than what has been recommended for SMI screening [2,15]. Furthermore, the patients’ coronary status was unknown in these studies, with no opportunity to evaluate the potential association between endothelial function and SMI according to the presence or the absence of CAD. This could be crucial as the patients with SMI but no CAD on angiography are likely to have functional vascular disorders such as abnormal coronary reserve or coronary endothelial dysfunction [16]. Finally, non-diabetic control subjects and non-diabetic overweight or obese patients were not included in these studies. If FMD has been shown to be lower in patients with type 2 diabetes than in age- and sex-matched subjects, BMI was higher in the former [17] and it is known that obesity is associated with endothelial dysfunction [18]. Thus, there is a need for further studies to validate the technique and evaluate the proper impact of diabetes instead of the combined effect of obesity with diabetes.

We raised the hypothesis that FMD would be impaired in the diabetic patients, with incremental impairment in those without SMI, those with SMI but no CAD and those with both SMI and CAD. The aim of the study was to investigate in a series of type 2 diabetic patients with a high cardiovascular risk according to the joint guidelines of the French Language Association for the Study of Diabetes and Metabolic Diseases (ALFEDIAM) and the French Society of Cardiology (SFC) if FMD was associated with SMI and/or asymptomatic CAD.

## Methods

### Participants

We recruited control subjects, non-diabetic obese subjects and type 2 diabetic patients in the Department of Diabetology of Jean Verdier Hospital (Bondy, France). The study was approved by the Ethical Committee of Aulnay-sous-Bois, France, and each enrolled patient gave informed consent (NCT00685984). Control subjects were free of known diabetes and had no cardiovascular risk factors. Obese subjects had neither known diabetes nor diabetes detected on oral glucose tolerance test nor any history of angina or myocardial infarction and had a normal resting ECG although they could have other cardiovascular risk factors. Eligibility criteria for type 2 diabetic patients included no history of myocardial infarction or angina pectoris, normal 12-lead resting ECG, and at least one of the ALFEDIAM-SFC criteria [2]: *(i)* patients over the age of 60 years or with diabetes for more than 10 years and with at least two or more of the following cardiovascular risk factors: dyslipidemia (total cholesterol > 6.5 mmol/l and/or LDL-cholesterol > 4.1 mmol/l, HDL-cholesterol < 0.9 mmol/l, triglycerides > 2.3 mmol/l and/or lipid lowering medication), hypertension (systolic/diastolic blood pressure ≥ 140/90 mmHg or anti-hypertensive therapy), active smoking or cessation for less than three years and major cardiovascular event before the age of 60 years in a first degree relative; *(ii)* patients, irrespective of their age or level of classical risk factors, presenting with either peripheral or carotid occlusive arterial disease (stenosis measured 50% by ultrasound examination performed in each patient) or macroproteinuria (urinary protein excretion rate ≥0.3 g/24 hours); *(iii)* patients, irrespective of their age, with microalbuminuria (urinary albumin excretion rate > 30 mg/day on at least two measurements) and at least two other classical risk factors; *(iv)* patients over the age of 45 years resuming sports activities after sedentary lifestyle. Exclusion criteria included congenital heart disease, pregnant women, congestive heart failure or known cardiopathy, renal insufficiency (creatinine clearance < 60 ml/min) and Raynaud syndrome.

### Cardiovascular investigations

#### Peripheral endothelial function

We evaluated FMD on the brachial artery as recommended by Coretti *et al.* Patients were explored in the supine position 24 hours after withdrawal of vasodilators and were instructed to avoid caffeine-containing products, smoking and exercise for at least twelve hours before the exploration [19]. Ultrasound images were obtained in the longitudinal plane using a high-resolution 10.0 MHz linear array transducer (Siemens Acuson sequoia C512). A blood pressure cuff was placed around the forearm, distal of the artery segment that was explored, and was inflated 50 mmHg above the patient’s systolic blood pressure for 5 minutes. A single investigator (IP) recorded the brachial artery diameter and flow velocity by 2D echography and pulsed Doppler at baseline, at deflation and 1 minute after deflation [19]. An angulation of the probe <60° was carefully sought. Analyses were performed off-line and blinded to test conditions and patient identity. Diameter measurements were done at telediastole. FMD was calculated as the percentage of increase (+) or decrease (-, which depicted paradoxical vasoconstriction) in the artery diameter from baseline to 1-minute after deflation. The diameter was calculated as the mean of at least 3 measurements manually performed with electronic calipers at end-diastole determined with simultaneous ECG recording. The intra-individual variability was tested in ten patients at one-hour interval. The intra-individual agreement index [20] was 0.04 and 0.04 for the artery diameter measurement at baseline and at 1 minute, respectively and repeatability coefficient was 0.09 cm and 0.06 cm respectively.

#### Screening for SMI

Each diabetic patient was planned to undergo both a myocardial scintigraphy and a stress echocardiography. The thallium 201 myocardial scintigraphy was performed after an ECG stress test, or a pharmacological stress test (dipyridamole injection), or both. The ECG stress test was performed in patients who could exercise on a bicycle ergometer and could be expected to have an interpretable exercise-ECG. When the patient was unable to exercise or when the ECG stress test result was indeterminate, a pharmacological stress test using dipyridamole was carried out [4,7,21]. Target heart rate was defined as 85% of maximal predicted heart rate (220-age). An abnormal scintigraphy imaging was defined as defects in at least 3 out of 17 segmental regions. Briefly, stress dobutamine echography was performed according to a protocol using 3-min stages with incremental dobutamine doses from 10 to 40 μg/kg/min and atropine, up to a total dose of 1 mg, as needed to increase the heart rate up to 85% of the predicted maximal heart rate. An abnormal stress dobutamine echocardiogram was defined by the presence of fixed akinetic or dyskinetic segment(s) and/or by the worsening or the development of a new wall motion abnormality, including a deterioration of wall motion after an initial improvement at low-dose dobutamine, in more than 2 concordant segments in a 17-segment model of the left ventricle. SMI was defined as an abnormal ECG stress test and/or abnormal myocardial scintiscan and/or abnormal stress echocardiogram.

#### Screening for CAD

A selective coronary angiography was performed in the diabetic patients with SMI within a period of 2 months after the noninvasive investigation. CAD was defined as a 70% narrowing of the luminal diameter in the left anterior descending artery, the circumflex artery, a well-developed marginal vessel or the right coronary artery, or as a 50% diameter narrowing of the left main coronary artery.

### Biochemical assays

The following measurements were recorded at the time of screening for SMI: HbA_1c_ (Dimension® technology, Siemens Healthcare Diagnosis Inc., Newark, USA), fasting glucose value measured on venous plasma by the glucose oxydase method (colorimetry, Kone Optima, Thermolab System), serum total cholesterol, HDL cholesterol and triglycerides (enzymatic colorimetry, Hitachi 912, Roche Diagnostic), creatininemia (colorimetry, Kone Optima, Thermolab System) and 24-hr urinary albumin excretion rate (laser immunonephelometry, BN100, Dade-Behring). The LDL cholesterol level was calculated according to the Friedwald formula and the creatinine clearance was assessed using the Modification of Diet in Renal Disease (MDRD) study’s formula. Vascular Cellular Adhesion Molecule (VCAM) was retrospectively measured according to the manufacturer’s instructions (quantikine ELISA kit, R&D Systems, Abingdon, UK), from samples stored at -80°C in the Biological Research Centre of our hospital. Each sample and standard protein was assayed in duplicate.

### Statistical analyses

Sample size was calculated in order to allow a sufficient power both to analyze differences between the different categories of diabetic patients and between diabetic patients and obese or control subjects. Preliminary studies [3,21,22] allowed considering that diabetic patients will be distributed in proportions 4/3/1 regarding the 3 categories: no SMI/SMI without CAD/SMI with CAD. Thus, considering a difference of 5% dilation of the brachial artery between the group with SMI and CAD and the 2 others and a standard deviation (SD) equal to 5%; a cohort of 120 diabetic patients will allow a power higher than 90% to detect a difference between the 3 groups using a two-sided 5% significance ANOVA level. In addition a sample size of 30 control subjects and 30 obese patients will allow a 90% power to detect a 6% difference between these groups and the diabetic patients.

According to their Gaussian or non-Gaussian statistical distribution, continuous variables were expressed as means ± SD or median (upper limits of first and third quartiles) and compared with parametric or non-parametric (Kruskall-Wallis) ANOVA for the three groups’ comparison and with either t-tests or Mann–Whitney tests with α-value adjusted for multiplicity by Bonferroni method for two-by-two comparisons. The significance of the differences in proportions was tested with the x^2^ test. Logistic regression was used for multivariate analyses based on models including *(i)* the factors that were associated with SMI (SMI-model 1) or asymptomatic CAD (CAD-model 1) with a p value ≤ 0.10 in univariate analyses, with in addition angiotensin conversion enzyme (ACE)-inhibitors and *(ii)* factors that have been previously reported to be associated with these conditions (model 2: age ≥60 years, diabetes duration ≥10 years, male gender, retinopathy, nephropathy, peripheral or carotid occlusive arterial disease, hypertension, dyslipidemia and smoking habits) [1-4].

The sensitivity, specificity, negative predictive value and positive predictive value of paradoxical vasoconstriction were assessed to diagnose SMI or silent CAD. Statistical analyses were carried out using SAS software, version 9.2 (SAS Institute). The 0.05 probability level was considered for statistical significance.

## Results

### Subjects

Twenty-five control subjects, 30 overweight or obese patients and 118 diabetic patients were included, whose characteristics are summarized in Table 1.

**Table 1:** Characteristics of control subjects, overweight or obese patients and diabetic patients

	**Control group**	**Overweight or obese patients**	**Diabetic patients**	**p**
	**n = 25**	**n = 30**	**n = 118**	
Age, years	23 (22;25)	37 (31;49)^*^	61 (56;67)^*†^	<0.01
Gender (male/female)	15/10	3/27^*^	72/46^†^	<0.01
Body mass index, kg/m^2^	21.7 (20.4;23.0)	34.1 (31.3;37.1)^*^	30.0 (26.9;34.7)^*†^	<0.01
Hypertension (%)	NA	7 (23.3)	103 (87.3)	<0.01
Dyslipidemia (%)	NA	12 (40.0)	103 (87.3)	<0.01
Total cholesterol, mmol/l		4.7 (4.4;5.5)	4.4 (3.8;5.2)	0.05
HDL cholesterol, mmol/l		1.21 (1.03;1.35)	1.08 (0.92;1.3)	0.06
LDL cholesterol, mmol/l		3.17 ± 0.90	2.65 ± 0.92	<0.01
Triglycerides, mmol/l		1.17 (0.90;1.79)	1.52 (1.10;2.20)	<0.05
Smoking (%)	NA	3 (10.0)	23 (19.5)	NS
FMD, %	3.95 (1.43;5.25)	4.25 (1.74;5.56)	0.61 (-1.22;3.2)^*†^	<0.01^§^
Paradoxical vasoconstriction (%)	3 (12)	3 (10.0)	47 (39.8)^*†^	<0.01^§^
VCAM, ng/ml		485 (435;656)	562 (430;677)	NS

Data are means ± SD or median (upper limits of first and third quartiles); *: p < 0.01 vs control subjects; †: p < 0.01 vs overweight or obese patients. ^§^: also after adjustment on age and gender.

FMD: flow mediated dilation, NA: non applicable, NS: non significant, VCAM: Vascular Cellular Adhesion Molecule.

### Flow mediated dilation measurements

FMD was measurable in all subjects and lower in the diabetic patients than in control subjects and in overweight or obese patients, with also a higher rate of paradoxical vasoconstriction (Table 1). This was also true after adjustment on age and gender.

In the diabetic patients, deflation cuff induced a mean increase in flow velocity of 288 (200;417)% and median FMD was 0.6 (-1.2;3.2)%. A paradoxical vasoconstriction was observed in 47 (39.8%) patients (Table 2) and associated with lower age (vasoconstriction vs no vasoconstriction: 59.1 ± 9.3 vs 62.5 ± 7.2 years respectively, p < 0.05) and HDL cholesterol levels (1.0 (0.8;1.2) vs 1.2 (1.0;1.3) mmol/l, p < 0.01); and with higher HbA1c (7.9 (7.0;9.6)%; 63 (53;81) mmol/mmol) vs 7.4 (6.7;8.4)%; 57 (50;68) mmol/mmol; p < 0.05) and triglyceride (1.8 (1.2;2.5) vs 1.4 (1.0;1.8) mmol/l, p < 0.05) levels. The patients without paradoxical vasoconstriction were also more likely to be treated with ACE-inhibitors (52.1 vs 31.9%, p < 0.05). There was no association between vasoconstriction and VCAM levels or urinary albumin excretion rate (Additional file 1: Table S1).

**Table 2:** Type 2 diabetic patients’ characteristics according to the presence or absence of silent myocardial ischemia

	**Total n = 118**	**No SMI n = 58**	**SMI n = 60**	**Univariate analysis**	Multivariate analysis*
				**p**	**Odds ratio [95CI] p**
**Clinical characteristics**					
Age ≥60 years (%)	72 (61.0)	39 (67.2)	33 (55)	NS	
Age, years	61.1 ± 8.2	62.1 ± 7.9	60.2 ± 8.5	NS	
Gender (Male/Female)	72/46	29/29	43/17	<0.05	NS
Body mass index, kg/m^2^	30.6 ± 5.4	31.6 ± 5.9	29.6 ± 4.7	<0.05	NS
**Diabetes**					
Diabetes duration, years	13 (10;20)	14 (9;21)	13 (11;19)	NS	
Diabetes duration ≥10 years (%)	90 (76.3)	42 (72.4)	48 (80)	NS	
HbA1c, %	7.5 (6.8;8.7)	7.5 (6.8;8.5)	7.7 (6.8;8.8)	NS	
HbA1c, mmol/l	58 (51;72)	58 (51;69)	61 (51;73)	NS	
Retinopathy (%)	58 (51.3)	25 (45.5)	33 (56.9)	NS	
Nephropathy (%)	49 (41.5)	18 (31.0)	31 (51.7)	<0.05	2.6 [1.2-5.7], p < 0.05
Creatinine clearance, ml/min	83 ± 22	84 ± 23	82 ± 21	NS	
Urinary albumin excretion rate (mg/day)	17 (7;97)	11(6;54)	32 (12;277)	NS	
Macroproteinuria (%)	25 (21.4)	9 (15.5)	16 (27.1)	NS	
Peripheral neuropathy (%)	54 (45.8)	24 (41.4)	30 (50.0)	NS	
**Additional cardiovascular risk factors:**					
Hypertension (%)	103 (87.3)	48 (82.8)	55 (91.7)	NS	
Anti-hypertensive treatment (%)	102 (86.4)	47 (81.0)	55 (91.7)	0.10	NS
Dyslipidemia (%)	103 (87.3)	48(82.8)	55(91.7)	NS	
HDL cholesterol, mmol/l	1.1 (0.9;1.3)	1.1 (1.0;1.3)	1.0 (0.8;1.3)	0.08	NS
Triglycerides, mmol/l	1.5 (1.1;2.2)	1.4 (1;1.8)	1.8 (1.1;2.4)	0.05	NS
LDL cholesterol, mmol/l	2.7 ± 0.9	2.5 ± 0.8	2.8 ± 1.0	NS	
Smoking (%)	23 (19.5)	9 (15.5)	14 (23.3)	NS	
Peripheral or carotid arterial disease (%)	17 (14.5)	8 (14)	9 (15)	NS	
VCAM, ng/ml	562 (430;677)	564 (464;665)	555 (412;683)	NS	
**Peripheral endothelial function:**					
Forearm mediated dilation, %	0.61 (-1.22;3.2)	1.64 (0;3.69)	0.12 (-2.3;1.58)	<0.01	
Paradoxical vasoconstriction (%)	47 (39.8)	17 (29.3)	30 (50.0)	<0.05	2.7 [1.2-5.9], p < 0.05
**Pharmacologic treatments**					
Statins (%)	88 (74.6)	41 (70.7)	47 (78.3)	NS	
Fibrates (%)	8 (6.8)	4 (6.9)	4 (6.7)	NS	
Platelet antiaggregants (%)	74 (62.7)	33 (56.9)	41 (68.3)	NS	
ACE-inhibitors (%)	52 (44.1)	25 (43.1)	27 (45)	NS	
ARBs (%)	52 (44.1)	24 (41.4)	28 (46.7)	NS	
Beta-blockers (%)	22 (18.6)	10 (17.2)	12 (20)	NS	
Calcium-channel blockers (%)	41 (34.7)	19 (32.8)	22 (36.7)	NS	
Sulfonylureas (%)	77 (65.3)	36 (62.1)	41 (68.3)	NS	
Metformin (%)	102 (86.4)	49 (84.5)	53 (88.3)	NS	
Thiazolidinediones (%)	33 (28.0)	14 (24.1)	19 (31.7)	NS	
Alpha-glucosidase inhibitors (%)	42 (35.6)	17 (29.3)	25 (41.7)	NS	
Insulin (%)	49 (41.5)	27 (46.6)	22 (36.7)	NS	

Data are means ± SD or median (upper limits of first and third quartiles); *: multivariate analysis taking into account the factors that were associated with silent myocardial ischemia with a p value ≤ 0.10 in univariate analyses (SMI-model 1).

95CI: 95% confidence interval, ACE-inhibitor: angiotensin conversion enzyme, ARB: angiotensin II receptor blocker, FMD: flow mediated dilation, NS: non significant, SMI: silent myocardial ischemia, VCAM: Vascular Cellular Adhesion Molecule.

### Screening for SMI and silent CAD in the diabetic patients

The characteristics of the 118 enrolled type 2 diabetic patients are further shown in Table 2. Regarding ALFEDIAM-SFC criteria, 47 (39.8%) of them fulfilled non exclusively the “type 2 diabetes with cardiovascular risk” criterion, 37 (31.4%) the “peripheral or carotid occlusive arterial disease or macroproteinuria” criterion, 18 (15.2%) the “microalbuminuria with cardiovascular risk” criterion, and 16 (15.4%) the “physical activity” criterion. Nephropathy as defined by microalbuminuria or macroproteinuria affected 49 patients.

A myocardial scintigraphy was performed in 109 patients including 56 after ECG stress test, 39 after dipyridamole injection and 14 after both, whereas it was unavailable or refused for 9 patients. Stress echographies were contributive in 90/118 patients, because of poor echogenicity in 28 patients. SMI was diagnosed in 60/118 (50.8%) patients according to an abnormal scintiscan (n = 24) or an abnormal stress echocardiogram (n = 25) or both (n = 11). A coronary angiography was subsequently performed in 59 of the 60 patients with SMI, and 21 of them (35.6%) had CAD, including one-vessel disease (n = 15), two- and three-vessel disease (n = 3 for both). A total of 81 patients had as planned both a myocardial scintigraphy and a stress echography. In these 81 patients, SMI was diagnosed in 44 patients according to abnormal scintigraphies in 13, abnormal stress echographies in 20 and both in 11 patients. A coronary angiography was subsequently performed in 43 of the 44 patients with SMI, and 17 of them (39.5%) had CAD.

### Association between peripheral flow mediated dilation and ischemic status in the diabetic patients

Baseline brachial artery diameter did not differ significantly in the 3 groups (patients without SMI: 3.68 ± 0.64 mm, with SMI but no CAD: 3.92 ± 0.64 mm; with SMI and CAD: 3.78 ± 0.54 mm). Deflation cuff induced an immediate and similar increase in flow velocity in the 3 groups (p = NS), as shown by the increase in velocities at cuff deflation: no SMI 299 (211;443); SMI but no CAD 290 (214;414); SMI and CAD 250 (150;413)%.

**Figure 1:**
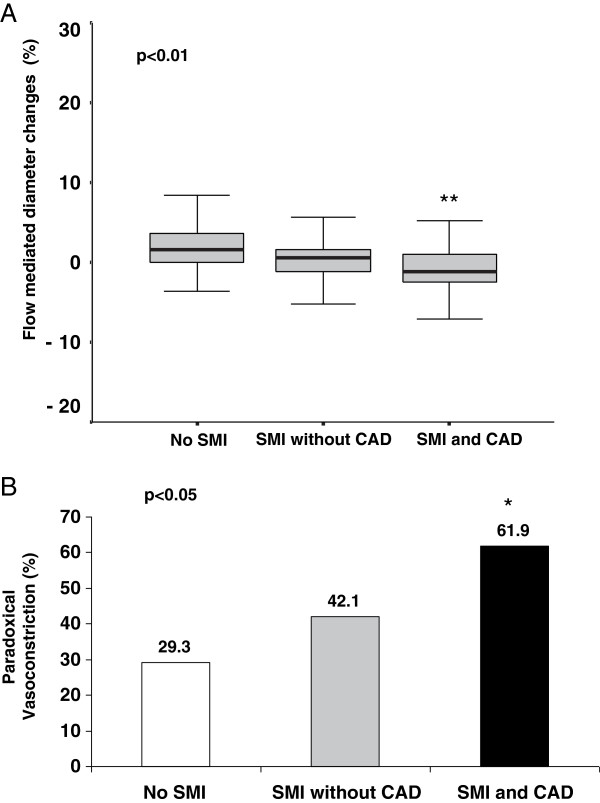
**Peripheral endothelial function according to silent myocardial ischemia and coronary artery disease status.** Flow mediated dilation **(Panel A)** and paradoxical vasoconstriction **(panel B)** in patients without silent myocardial ischemia (no SMI), with SMI but no coronary artery disease (SMI, no CAD) and with CAD (SMI and CAD). * p < 0.05 and ** p < 0.01 vs patients without SMI.

FMD was more impaired and the prevalence of paradoxical vasoconstriction was higher in the diabetic patients with SMI than in those without (Table 2) and in the patients with asymptomatic CAD than in those without (Table 3). Figure 1 shows the values of FMD (panel A) and the prevalence of paradoxical vasoconstriction (panel B) in the patients without SMI, those with SMI but no CAD, and those with both SMI and CAD. The results were similar when only the 81 patients having had both myocardial scintigraphy and stress echography were considered: FMD 1.63 (0.00;3.59), 0.46 (-1.22;1.61) and -1.22 (-3.8;0.25) (p < 0.01); paradoxical vasoconstriction 27.0, 42.3 and 64.7%, (p < 0.05), in patients without SMI, with SMI but no CAD and with both SMI and CAD, respectively.

**Table 3:** Patients’ characteristics according to the presence or absence of asymptomatic coronary artery disease in the diabetic cohort

	**No CAD n = 96**	**CAD n = 21**	**Univariate analysis**	Multivariate analysis*
			**p**	**Odds ratio [95CI] p**
**Clinical characteristics:**				
Age, years	61.0 ± 8.1	62.0 ± 8.8	NS	
Gender (Male/Female)	56/40	16/5	NS	
Body mass index, kg/m^2^	31.0 ± 5.6	28.6 ± 3.9	0.07	NS
**Diabetes:**				
Diabetes duration, years	14 (10;21)	12 (11;19)	NS	
HbA1c, %	7.5 (6.8;8.9)	7.7 (6.9;8.0)	NS	
HbA1c, mmol/mmol	58 (51;74)	61 (52;64)	NS	
Retinopathy (%)	47 (51.1)	11 (55.0)	NS	
Nephropathy (%)	38 (39.6)	11 (52.4)	NS	
Creatinine clearance, ml/min	83.6 ± 21.6	79.8 ± 24.2	NS	
Urinary albumin excretion rate (mg/day)	14 (7;74)	32 (7;495)	NS	
Macroproteinuria (%)	18 (18.9)	7 (33.3)	NS	
Peripheral neuropathy (%)	45 (46.9)	9 (42.9)	NS	
**Additional cardiovascular risk factors:**				
Hypertension (%)	83 (86.5)	19 (90.5)	NS	
Anti-hypertensive treatment (%)	82 (85.4)	19 (90.5)	NS	
Dyslipidemia (%)	83 (86.5)	19 (90.5)	NS	
HDL cholesterol, mmol/l	1.1 (1.0;1.3)	1.0 (0.8;1.0)	0.08	NS
Triglycerides, mmol/l	1.5 (1.1;2.1)	2.0 (1.4;2.5)	<0.05	NS
LDL cholesterol, mmol/l	2.6 ± 0.8	3.0 ± 1.2	0.10	NS
Smoking (%)	18 (18.8)	5 (23.8)	NS	
VCAM, ng/ml	549 (422;665)	621 (526;712)	NS	
**Peripheral endothelial function:**				
Forearm mediated dilation, %	1.13 (-0.4;3.28)	-1.22 (-2.5;1.0)	<0.01	
Paradoxical vasoconstriction (%)	33 (34.4)	13 (61.9)	<0.05	3.1 [1.2-8.2], p < 0.05
**Pharmacologic treatments**				
Statins (%)	72 (75)	15 (71.4)	NS	
Fibrates (%)	5 (5.2)	3 (14.3)	NS	
Platelet antiaggregants (%)	58 (60.4)	15 (71.4)	NS	
ACE-inhibitors (%)	46 (47.9)	6 (28.6)	NS	
ARBs (%)	39 (40.6)	12 (57.1)	NS	
Beta-blockers (%)	17 (17.7)	5 (23.8)	NS	
Calcium-channel blockers (%)	31 (32.3)	10 (47.6)	NS	
Sulfonylureas (%)	65 (67.7)	11 (52.4)	NS	
Metformin (%)	83 (86.5)	18 (85.7)	NS	
Thiazolidinediones (%)	26 (27.1)	7 (33.3)	NS	
Alpha-glucosidase inhibitors (%)	38 (39.6)	4 (19)	0.08	NS
Insulin (%)	38 (39.6)	10 (47.6)	NS	

Data are means ± SD or median (upper limits of first and third quartiles); *: multivariate analysis taking into account the factors that were associated with coronary artery disease with a p value ≤ 0.10 in univariate analyses (CAD-model 1).

95CI: 95% confidence interval, ACE-inhibitor: angiotensin conversion enzyme, ARB: angiotensin II receptor blocker, CAD: coronary artery disease, FMD: flow mediated dilation, NS: non significant, VCAM: Vascular Cellular Adhesion Molecule.

### Factors associated with SMI and asymptomatic CAD

The factors associated with SMI were male gender, body mass index, nephropathy, triglycerides levels, FMD and paradoxical vasoconstriction (p < 0.05) with a trend for HDL-cholesterol (p < 0.10) (Table 2). To explain SMI, multiple logistic regression analyses were performed with these parameters (SMI-model 1) and paradoxical vasoconstriction. SMI was independently associated with paradoxical vasoconstriction and nephropathy (Table 2). When FMD instead of paradoxical vasoconstriction was considered in the same model, FMD (odds ratio 0.86 [95% confidence interval 0.77-0.96], p < 0.01) and nephropathy (OR 2.57 [1.17-5.63], p < 0.05) were independently associated with SMI. The results were also similar when treatment with ACE-inhibitors was added to SMI-model 1. FMD or paradoxical vasoconstriction was also independently associated with SMI when the analyses were restricted to the 81 patients having had both the myocardial scintigraphy and the stress echography (data not shown). With model 2 considering the factors previously associated with silent coronary status, the results both for paradoxical vasoconstriction and FMD were similar as the ones of SMI-model 1. Paradoxical vasoconstriction had a sensitivity of 50.0%, a specificity of 70.7%, and positive and negative predictive values of respectively 63.8% and 57.7%, to detect SMI.

The factors associated with asymptomatic CAD were triglyceride levels, FMD and paradoxical vasoconstriction, with a trend for body mass index, HDL and LDL cholesterol levels and treatment with alpha glucosidase inhibitors (Table 3). To explain asymptomatic CAD, a multiple logistic regression analysis was performed with these parameters (CAD-model 1) and paradoxical vasoconstriction or FMD. CAD was associated only with paradoxical vasoconstriction (Table 3) or with FMD (OR 0.83 [0.73-0.95], p < 0.01). The results were similar when only the 81 patients having had both the myocardial scintigraphy and the stress echography were considered in CAD-model 1, for paradoxical vasoconstriction (OR 3.49 [1.07;11.44], p < 0.05) or FMD (OR 0.84 [0.72; 0.98], p < 0.05). The results of multivariate analyses considering the parameters included in model 2 with paradoxical vasoconstriction or FMD were similar. Paradoxical vasoconstriction had a sensitivity of 61.9%, a specificity of 65.6%, and positive and negative predictive values of respectively 28.3% and 88.7%, to detect asymptomatic CAD.

## Discussion

We confirmed that peripheral FMD is impaired in type 2 diabetes, as compared to control subjects and non-diabetic obese patients. In particular, we report here for the first time that FMD is independently associated with SMI; and even more specifically when ischemia is associated with CAD on angiography. This was observed while the immediate post-ischemic flow increase, a surrogate for microcirculation, remained similar whatever the heart ischemic status in these diabetic patients.

In the present study, the mean FMD was around 4% in control subjects and overweight/obese patients, and 1% in diabetic patients. The fact that FMD was lower in diabetic patients than in non-diabetic obese subjects shows that obesity *per se* did not affect FMD and that diabetes plays a major role in the impairment of FMD. FMD was lower than the levels reported elsewhere. Indeed, the normal values of FMD have not been well-established in a control population and are widely variable according to methods for measurements [19]. Bots *et al.* have reviewed more than 200 papers from 1992 to 2001 and reported in healthy subjects FMD from 0.2 to 19.2% and in diabetic patients from 0.75 to 12% [23]. In their conclusion technical aspects of measurements, location and duration of occlusion may explain some of the differences while type of equipment, location of measurement and occlusion pressure do not. Our local control groups had low FMD but our results in our diabetic cohort may also be explained by long standing diabetes and a high *a priori* cardiovascular risk, due to our inclusion criteria. For example, the prevalence of asymptomatic CAD was high in our cohort (17.8%), whereas we previously reported a lower prevalence (10-15%) in patients with type 2 diabetes who were screened for SMI only by stress scintigraphy [7,21,22] instead of two tests in the present study. FMD may be considered as an integrator of cardiovascular risk, *i.e.* a marker of cardiovascular stress related to the presence of risk factors and their levels, whatever the underlying mechanism. In the present study, paradoxical vasoconstriction was associated with a more impaired lipid profile, a poorer glycemic control, less current treatment by ACE-inhibitors, and a trend for smoking, despite a lower age.

FMD studies explore the peripheral vasculature response to transient ischemia. The flow response, as depicted by the flow velocity increase after cuff deflation, reflects the distal microcirculation response to ischemia, and an impaired response has been reported to predict cardio-vascular events [24]. Diameter changes after the increase in flow depend on the endothelium, mainly through a nitric oxide-dependent mechanism, but also on vascular smooth muscle cell contraction/relaxation. Endothelial dysfunction may be considered as a cardiovascular risk factor or at least as a cardiovascular risk marker [12,25], but impairment of vascular smooth muscle cell function has been also reported in diabetic patients [9] and may be involved in altered FMD. The mechanism of impaired FMD cannot be explained in our results since we did not test specifically vascular smooth muscle cell function, like Peix *et al.*[14]. The absence of significant difference between patients who constricted their brachial artery and those who did not for VCAM and albuminuria, which are usually considered as endothelium markers [26], might be consistent with the role of impaired smooth muscle cell function in our population. The important fact is that an impaired FMD response *per se*, whatever its mechanism, was shown to predict a poor cardio-vascular prognosis in several large population studies [25,27].

Abnormal coronary vasomotion [10,28] and coronary endothelial dysfunction [29] are also associated with a poor cardiovascular prognosis in diabetes. A direct relationship between peripheral and coronary vascular function may be difficult to demonstrate. FMD was reported to statistically correlate with coronary response to acetylcholine, but this correlation was weak [30]. Gori *et al.* recently showed that using FMD provides significant additional information in predicting the presence of CAD in patients suffering from angina [31]. Furthermore, FMD has recently been shown to be independently associated with slow coronary flow in patients with angina and non significant narrowed CAD [32]. Although, we have clearly demonstrated here that an altered FMD was independently associated with SMI, other studies failed to show a strong association between FMD and SMI. This discrepancy seems to be related to the difference in the cardiovascular risks of the patients in these studies compared to our study population (Table 2: diabetes duration 13 years, hypertension and dyslipidemia in 87.3% and smoking in 19% of the patients). In the Detection of Ischemia in Asymptomatic Diabetics (DIAD) study, FMD was measured in 75 asymptomatic type 2 diabetic patients and was found to be similar in those with or without SMI [13]. The cardiovascular risk profile was better than in our study population, with a mean diabetes duration of 8.4 years, and hypertension, dyslipidemia and smoking habits in 49%, 59% and 8% of the patients, respectively, and only 15 (20%) of the patients had SMI. When the cardiovascular risk profile of the patients was intermediate as in Peix *et al*. study (diabetes duration 11 years, age 58 years, hypertension, dyslipidemia and smoking habits in 77%, 73% and 32% of the patients, respectively), there was a higher prevalence of abnormal FMD in those with SMI as compared with those without, whereas no difference was found for the mean values of FMD [14]. As reported by Naka *et al.*, duration of diabetes appears to be an important factor for developing impaired FMD [33]. In these two studies performed in diabetic patients, coronary status was not determined by angiography. This is a crucial issue as SMI actually includes two entities: only 30-70% of the patients with SMI have significant CAD [3] while ischemia in patients without CAD may result from functional disorders [16], such as abnormal coronary reserve or coronary endothelial dysfunction. In our study population, 35% of the patients with SMI had CAD. We report for the first time that the flow-mediated vascular response was worse, with more paradoxical vasoconstriction, when SMI or CAD were present. Abnormal FMD was gradually further impaired in the patients without SMI, with SMI but no CAD, and with both SMI and CAD (Figure 1). This result is consistent with the role of silent coronary disease in the poorer prognosis associated with lower FMD in the diabetic population.

The use of FMD as a screening test for SMI was tested in the DIAD study [13]. The negative predictive value for SMI of a normal FMD was 93%. FMD was considered as abnormal when <8% *i.e.* at the threshold that maximized the negative predictive value and had the least impact on sensitivity while the study did not include control subjects. The threshold that we considered in the present study was lower (<0%, *i.e.* paradoxical vasoconstriction) and was in line with the high cardiovascular risk of our patients. We found that paradoxical vasoconstriction was independently associated not only with SMI but also with CAD. However, while the presence of a paradoxical vasoconstriction had an 88.7% negative predictive value for CAD, the positive predictive value for CAD was too low to suggest the inclusion of this criterion in the algorithm of CAD screening.

The present study has some limitations. The control groups were not matched for gender, and by definition age and BMI were different in the three groups of patients (healthy controls, non-diabetic obese patients and diabetic patients). However, FMD was lower in the diabetic patients even after adjustment on age and gender. Due to obvious ethical issues, no coronary angiography was performed in the patients without SMI, and some patients with CAD but no SMI may have been missed. Furthermore, the cut-off for significant epicardial CAD we used was 70% stenoses, whereas stenoses are nowadays considered as significant with milder stenoses (i.e. 50% or more) when abnormal fractional flow reserve is observed. However, in our study, these measurements were not available for all the patients. Our FMD cutoff of 0% may not be applicable to diabetic patients with a low *a priori* cardiovascular risk. Lastly, we could not distinguish whether abnormal FMD resulted from endothelium-dependent or -independent disorders as nitroglycerin-induced vasodilation was not tested.

## Conclusions

Our results show in a cohort of asymptomatic type 2 diabetic patients with a high cardiovascular risk that impaired forearm FMD with a paradoxical vasoconstriction is associated with SMI and CAD. However the present data cannot lead to suggest FMD as a screening test for silent CAD.

## Abbreviations

ACE-inhibitor: Angiotensin conversion enzyme; ALFEDIAM-SFC: French Language Association for the Study of Diabetes and Metabolic Diseases (ALFEDIAM), and French Society of Cardiology (SFC: Société Française de Cardiologie); ARB: Angiotensin II receptor blocker; AROC: Area under the receiver operating characteristic curve; CAD: Coronary artery disease; DIAD: Detection of Ischemia in Asymptomatic Diabetics; FMD: Flow-mediated dilation; SMI: Silent myocardial ischemia; VCAM: Vascular cellular adhesion molecule.

## Competing interests

The authors declare no competing interests.

## Authors’ contributions

MTN researched data, made statistic, contributed to discussion; IP performed the FMD and stress echography studies, contributed to discussion, reviewed/edited manuscript; PV directed research, contributed to discussion, reviewed/edited manuscript; HR made statistic; EV made statistic, contributed to discussion, reviewed/edited manuscript; CLM made biochemical measurement and contributed to discussion, AN contributed to discussion; EC directed research, wrote manuscript. Prof Eric Vicaut is the guarantor of this work and, as such, had full access to all the data in the study and takes responsibility for the integrity of the data and the accuracy of the data analysis. All authors read and approved the final manuscript.

## Supplementary Material

Additional file 1: Table S1 Type 2 diabetic patients’ characteristics according to the presence or absence of paradoxical vasoconstriction. Click here for file
