# Supplementary material for: Flow-mediated-paradoxical vasoconstriction is independently associated with asymptomatic myocardial ischemia and coronary artery disease in type 2 diabetic patients
Source: Cardiovasc Diabetol. 2014 Jan 15;13:20. doi: 10.1186/1475-2840-13-20 (PMC3901336; doi:10.1186/1475-2840-13-20)
Supplement: Additional file 1: Table S1 — Type 2 diabetic patients’ characteristics according to the presence or absence of paradoxical vasoconstriction. [file 1475-2840-13-20-S1.doc]

**On-line additional Table: Type 2 diabetic patients’ characteristics according to the presence or absence of paradoxical vasoconstriction**

|  | Total | No vasoconstriction | Vasoconstriction | p |
| --- | --- | --- | --- | --- |
|  | n=118 | n=71 | n= 47 |  |
| **Clinical characteristics** |  |  |  |  |
| Age, years | 61.1±8.2 | 62.5±7.2 | 59.1±9.3 | <0.05 |
| Age ≥60 years | 72 (61.0) | 50 (70.4) | 22 (46.8) | <0.05 |
| Gender (Male/Female) | 72/46 | 40/31 | 32/15 | NS |
| Body mass index, kg/m² | 30.6±5.4 | 30.8±5.5 | 30.3±5.3 | NS |
| **Diabetes:** |  |  |  |  |
| Diabetes duration, years | 13 (10;20) | 13 (10;19) | 14 (9;21) | NS |
| Diabetes duration ≥10 | 90 (76.3) | 55 (77.5) | 35 (74.5) | NS |
| HbA1c, % | 7.5 (6.8;8.7) | 7.4 (6.7;8.4) | 7.9 (7;9.6) | <0.05 |
| HbA1c, mmol/l | 58 (51;71) | 57 (50;68) | 63 (53;81 | <0.05 |
| Retinopathy (%) | 58 (51.3) | 35 (52.2) | 23 (50.0) | NS |
| Nephropathy (%) | 49 (41.5) | 31 (43.7) | 18 (38.3) | NS |
| Creatinine clearance, ml/min | 82.8±22.0 | 84.6±20.3 | 80.1±24.2 | NS |
| Urinary albumin excretion rate (mg/day)y) | 16.9 (7.2;97.5) | 18.7 (7.2;74.1) | 13.5 (6.8;209.1) | NS |
| Macroproteinuria (%) | 25 (21.4) | 12 (17.1) | 13 (27.7) | NS |
| Peripheral neuropathy (%) | 54 (45.8) | 34 (47.9) | 20 (42.6) | NS |
| Peripheral or carotid arterial disease | 17 (14.5) | 8 (11.4) | 9 (19.1) | NS |
| **Additional cardiovascular risk factors** |  |  |  |  |
| Hypertension (%) | 103 (87.3) | 60 (84.5) | 43 (91.5) | NS |
| Systolic blood pressure, mmHg | 131±17 | 131±17 | 131±17 | NS |
| Diastolic blood pressure, mmHg | 70 (68;80) | 70 (69;80) | 73 (65;80) | NS |
| Anti-hypertensive treatment (%) | 102 (86.4) | 60 (84.5) | 42 (89.4) | NS |
| Dyslipidemia (%) | 103 (87.3) | 61 (85.9) | 42 (89.4) | NS |
| HDL cholesterol, mmol/l | 1.1 (0.9;1.3) | 1.2 (1.0;1.3) | 1.0 (0.8;1.2) | <0.01 |
| Triglycerides, mmol/l | 1.5 (1.1;2.2) | 1.4 (1.0;1.8) | 1.8 (1.2;2.5) | <0.05 |
| LDL cholesterol, mmol/l | 2.7±0.9 | 2.6±1.0 | 2.7±1.0 | NS |
| Smoking (%) | 23 (19.5) | 10 (14.1) | 13 (27.7) | 0.07 |
| VCAM, ng/ml | 562 (430;677) | 549(450;657) | 586(428;712) | NS |
| **Pharmacologic treatments** |  |  |  |  |
| Statins (%) | 88 (74.6) | 54 (76.1) | 34 (72.3) | NS |
| Fibrates (%) | 8 (6.8) | 2 (2.8) | 6 (12.8) | 0.06 |
| Platelet antiaggregants (%) | 74 (62.7) | 46 (64.8) | 28 (59.6) | NS |
| ACE-inhibitors (%) | 52 (44.1) | 37 (52.1) | 15 (31.9) | <0.05 |
| ARBs (%) | 52 (44.1) | 28 (39.4) | 24 (51.1) | NS |
| Beta-blockers (%) | 22 (18.6) | 11 (15.5) | 11 (23.4) | NS |
| Calcium-channel blockers (%) | 41 (34.7) | 20 (28.2) | 21 (44.7) | 0.07 |
| Sulfonylureas (%) | 77 (65.3) | 43 (60.6) | 34 (72.3) | NS |
| Metformin (%) | 102 (86.4) | 62 (87.3) | 40 (85.1) | NS |
| Thiazolidinediones (%) | 33 (28.0) | 16 (22.5) | 17 (36.2) | NS |
| Alpha-glucosidase inhibitors (%) | 42 (35.6) | 28 (39.4) | 14 (29.8) | NS |
| Insulin (%) | 49 (41.5) | 27 (38) | 22 (46.8) | NS |

Data are means ± SD or median (upper limits of first and third quartiles);

ACE-inhibitor: angiotensin conversion enzyme, ARB: angiotensin II receptor blocker, FMD: flow mediated dilation, NS: non significant, VCAM: Vascular Cellular Adhesion Molecule
